# Stainless steel and NiTi torque archwires and apical root resorption

**DOI:** 10.1007/s00056-020-00244-4

**Published:** 2020-09-01

**Authors:** Andrea Wichelhaus, Marc Dulla, Hisham Sabbagh, Uwe Baumert, Thomas Stocker

**Affiliations:** Department of Orthodontics and Dentofacial Orthopedics, University Hospital, LMU Munich, Goethestraße 70, 80336 Munich, Germany

**Keywords:** Incisors, Orthodontic therapy, Tooth movement, Root-crown ratio, Orthodontic appliances, Frontzähne, Kieferorthopädische Therapie, Zahnbewegung, Wurzel-Kronen-Verhältnis, Orthodontische Apparaturen

## Abstract

**Objective:**

The amount of apical root resorption when using the torque-segmented archwire (TSA) was investigated as well as the extent and direction of the therapeutically indicated apical movement and the treatment duration.

**Materials and methods:**

The degree of apical root resorption in 18 randomly chosen Class II and Class I patients treated with the TSA, as well as in 18 conventionally treated patients were evaluated using pre- and posttreatment panoramic radiographs. The sagittal and vertical apical movements and inclination changes were determined based on pre- and posttreatment lateral cephalograms. Nonparametric tests were applied to test between treatment groups and steps. The Mann–Whitney U test, Kruskal–Wallis, Pearson correlation and Wilcoxon signed-rank test were applied for statistical analysis (*p* < 0.05).

**Results:**

The incidence of root resorptions was 89–94.4% in low or moderate level. The relative root–crown ratio (rRCR) was not statistically different between the TSA and control groups except tooth 12. The axis of the incisors in the TSA group was significantly improved. The main direction of movement of the apices of the central incisors was retrusion and extrusion. No interdependence between the amount of resorption and the parameters of treatment duration, extent and direction of apical movement were found.

**Conclusion:**

The results of the study showed that the amount of apical root resorption with the TSA is slight to moderate and can be compared to conventional orthodontic treatment. The TSA is hence a suitable method for applying targeted torques to the incisors.

## Abstract (de)

**Ziel:**

Der Umfang der apikalen Wurzelresorption ebenso wie das Ausmaß und die Richtung der therapeutisch angenommenen apikalen Bewegung sowie die Behandlungsdauer wurden bei Anwendung des Torquesegmentbogens (TSB) untersucht.

**Methoden:**

Das Ausmaß der apikalen Wurzelresorption wurde an 18 willkürlich ausgewählten Klasse-II- und -I-Patienten, die mit dem TSB behandelt wurden, und an 18 konventionell behandelten Patienten anhand von prä- und posttherapeutisch erstellten Panoramaschichtaufnahmen beurteilt. Die sagittalen und vertikalen apikalen Bewegungen sowie die Änderungen der Inklination wurden anhand der FRS(Fernröntgenseitenbilder)-Aufnahmen bestimmt. Unterschiede zwischen Behandlungsgruppen und -schritten wurden mit nichtparametrischen Tests bestimmt. Mann-Whitney-U-, Kruskal-Wallis‑, Pearson-Korrelation- und Wilcoxon-Rangsummen-Tests wurden zur statischen Analyse verwendet (*p* < 0.05).

**Ergebnisse:**

Die Inzidenz der Wurzelresorption betrug 89–94,4 % für geringes bis moderates Ausmaß. Es gab keinen statistisch signifikanten rRCR(„relative root–crown ratio“)-Unterschied zwischen der TSB- und der Kontrollgruppe bis auf Zahn 12. Die Inzisivusachse in der TSB-Gruppe wurde signifikant verbessert. Die Hauptbewegungsrichtung der Apices der zentralen Schneidezähne waren Retrusion und Extrusion. Es fand sich keine Interdependenz zwischen dem Umfang der Resorption und den Parametern Behandlungsdauer, Ausmaß und Richtung der apikalen Bewegung.

**Schlussfolgerung:**

Die Studienergebnisse zeigen, dass der Umfang der apikalen Wurzelresorption mit TSB leicht bis moderat ist und mit der konventionellen orthodontischen Behandlung vergleichbar. Der TSB ist daher geeignet, Torque auf die Schneidezähne zu übertragen.

## Introduction

The root torque of the anterior teeth is an important tool in orthodontic treatment. The buccopalatal angulation of the root known as palatal root torque in orthodontics is an important step in creating functionally correct static and dynamic occlusal relationships and guaranteeing good support to the anterior teeth. Bodily retraction of the anterior teeth and simultaneous application of torque are often necessary, especially in the course of premolar extraction treatment.

For correct axial adjustment of the roots of the incisors, torque must be transferred to the teeth via orthodontic appliances. The torque angle in the bracket when using the straight wire technique does not transfer suitable torques in many cases because of slot geometry [53]. In these cases, a defined, biomechanically effective torque therefore needs to be produced via torsion of wires. The torques dependent on applied force and range between 15 and 20 Nmm as recommended in the literature [5] for all four upper incisors [55]. However, in some instances torques of around 5 Nmm are also specified [39]. These values ascertained from the literature are a guide and are dependent on the root geometry of the teeth or the biological circumstances in the individual patient. If forces and torques are biomechanically uncontrolled or too high, the risk of apical root resorptions will increase [10, 13, 34, 45]. In addition to the force and torque magnitude, the duration of force/torque application, force direction, treatment mechanics and treatment period should be noted as influencing variables in relation to resorption [2, 17, 23, 28, 31, 33, 34]. Torque movement of the apex causes a local concentration of pressure at the root tip and can reach a fourfold level compared with pure translatory tooth movements [47]. However, root apex resorptions are dependent on genetic and biological factors as well as the effects of the orthodontic treatment [51].

**Fig. 1:**
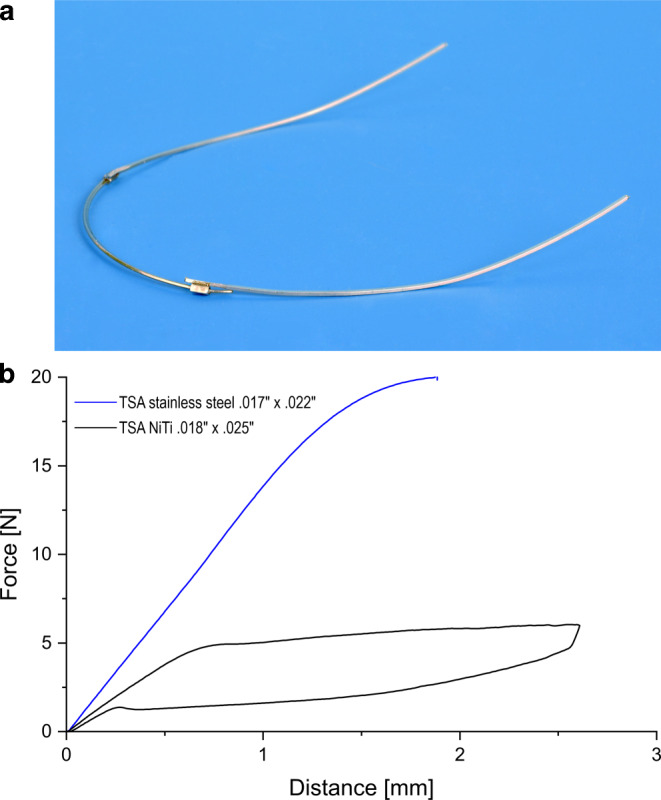
**a** Torque segmented archwire (TSA) with 1st (horizontal contouring), 2nd (sweep) and 3rd order bends (buccal root torque). **b** Mechanical characteristics of the individual parts of an TSA. Stainless steel shows Hookean behavior, whereas the NiTi alloy part exhibits the typical hysteresis curve **a** Torquesegmentbogen (TSA) mit Biegungen 1. (horizontale Konturierung), 2. (Sweep) und 3. Ordnung (bukkaler Wurzeltorque). **b** Mechanische Eigenschaften der individuellen Bestandteile eines TSA. Der Edelstahlbogen zeigt Hooksches Verhalten, während die NiTi-Komponente eine typische Hysteresekurve erzeugt

Nickel–titanium alloys (NiTi) are particularly well suited to transfer small forces and torques to the teeth because of their low Young’s modulus [6, 54]. In addition, the alloy is characterized by its superelastic material behavior. However, this behavior only arises when the material is adequately stretched or loaded. As a result, defined forces and torques can be orthodontically applied as well as small forces and torques. To utilize the superelasticity of NiTi alloy in order to change incisors’ root torque a segment of a stainless steel archwire was replaced with NiTi alloy. This combination provides the rigidness and stability of stainless steel on the “side-parts” on the one hand, as well as the small forces and torques produced by the superelastic NiTi alloy on the other hand. Torque segmented archwires (TSA), which were developed specifically for this purpose [47, 55], are available in prefabricated form (Forestadent, Pforzheim, Germany). The TSA consists of a pretorqued superelastic archwire component (NiTi) for the incisors and two steel segments attached via a clamp connection for the posterior teeth (Fig. 1a). If the mechanical load-deflection behavior of the entire TSA compound is measured as published by Wichelhaus and Sander [55], the resulting curve shows an incomplete superelasticity even though the frontal segment is made from superelastic material. This can be attributed to the fact that it is not possible to separate the NiTi hysteretic behavior from the linear elasticity of the stainless steel component. However, it has to be considered that the mechanical performance of the entire TSA compound is clinically almost irrelevant because the main stresses and moments are generated in the interbracket segments which are made of either stainless steel or NiTi. To prove the superelastic character of the TSA’s NiTi part, three-point bending measurements were conducted (Fig. 1b).

As apical root resorptions are a very commonly occurrence with the upper incisors [2, 46], the aim of the present study in using the TSA was to investigate the amount of apical root resorption compared with conventional orthodontic appliances. Furthermore, the extent and direction of the therapeutically indicated apical movement and the treatment duration were also evaluated.

## Materials and methods

### Patient groups

In a randomized retrospective follow-up study, we investigated the effect of TSA versus conventional orthodontic appliance application on the amount of apical root resorption. As such, the study group comprised of 18 randomly chosen successfully treated Class I and Class II patients (11 female, 7 male) from the Department of Orthodontics and Pediatric Medicine of Basel University and included 7 extraction cases. The complementary control group consisted of 18 randomly chosen Class I and Class II patients (11 female, 7 male) from the Department of Orthodontics and Dentofacial Orthopedics of the University of Munich and included 10 extraction cases. In all cases, there was a therapeutic necessity to apply torque on the maxillary incisors. In our treatment concept, torque with the stainless steel wire is not applied in patients with critical anchorage. In this case, we prefer the TSA because the sagittal force is lower in comparison to the stainless steel arch wire. Both methods apply torque changes to the maxillary incisors. The local ethics committee approved the study protocol (project number 19-815). On average, torque treatment of the patients in the study group started at 18.6 ± 7.5 years of age (range 11.4–37.3 years) and lasted for 105 ± 44 days. Within the control group patient torque treatment started at 14.8 ± 1.8 years of age (range 12.6–18.9 years). These patients were treated for 146 ± 52 days.

The torque application by means of the TSA took place using a standardized archwire sequence: 0.014″ NiTi, 0.016″ NiTi, 0.016″ × 0.022″ NiTi, 0.016″ × 0.022″ stainless steel, TSA (0.018″ × 0.025″ NiTi with 45° pretorque) and 0.018″ × 0.025′ stainless steel in the 0.022″ slot technique with MBT prescription. The effective torque in the 0.018″ × 0.025″ wire dimension is about 2° [11]. If sweep is necessary, an additional torque of 15° occurs. If no sweep is necessary, a torque of 30° was bent into the wire applying a moment of 20 Nmm [8].

The tooth axis in relation to the occlusal plane was clinically controlled with a special torque key [55]. The archwire sequence applied in the control group was identical to the one previously described, but without TSA.

### Examination of radiographs

For all patients lateral cephalograms and panoramic radiographs taken before and after treatment were examined. Because of the retrospective character of the study, no specific fixation devices were used for X‑rays, and the X‑rays taken routinely for diagnostics were used for the evaluation in this study.

**Fig. 2:**
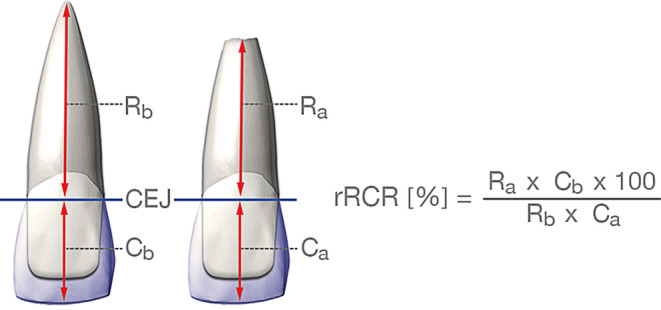
Assessment of pre- and posttreatment crown (C_x_) and root (R_x_) length (*CEJ* cementoenamel junction) according to Fritz et al. [15]. Individual relative root–crown ratios (rRCR) were calculated using pre- and posttreatment root and crown length using the given equation Bestimmung der Kronen- und Wurzellängen (C_x_ und R_x_) prä- und posttherapeutisch (*CEJ *Schmelz-Zement-Grenze) nach Fritz et al. [15]. Die Berechnung der individuellen relativen Wurzellängenänderung (rRCR) erfolgte gemäß der dargestellten Formel

For each of the examined panoramic radiographs root and crown length of the central and lateral incisors were measured according to the method presented by Linge and Linge [27] and Fritz et al. [15]. The distance between the incisal edge and the cementoenamel junction represented the crown length, correspondingly the distance between the cementoenamel junction and the apex represented the root length (Fig. 2). Because the crown length should not change during treatment, the proportion between crown and root length should stay constant in different radiographs of the same teeth at the same time. This allows to rate the radiographs widely independently of projections, recording angles or nonstandardized conditions [15, 26]. Using these measures, the root-to-crown-ratio (RCR) before and after treatment for each of the examined teeth was calculated (Fig. 2). The relative root-to-crown ratio (rRCR) (Fig. 2, formula) is the quotient of the RCR values of a given tooth before and after treatment and, thus, reflects the amount of root resorption [15, 16, 26]. As such, a rRCR ≥100% means “no resorption” and rRCR values <100% signify root resorption.

**Fig. 3:**
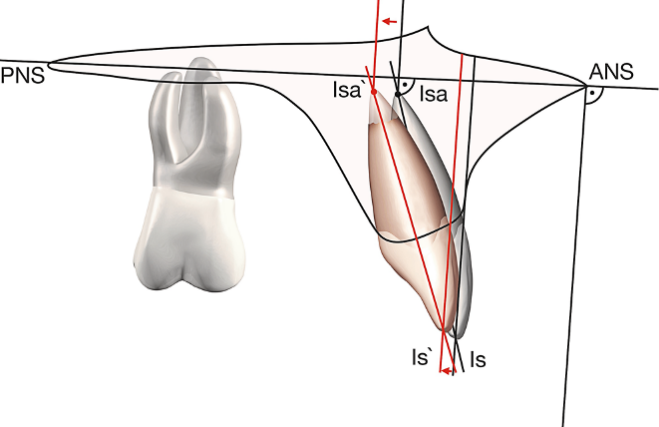
Analysis of the amount of movement of the apex based on pre- and posttreatment lateral cephalograms. Sagittal and vertical movements (mm) were determined. *ANS* anterior nasal spine, *PNS* posterior nasal spine, *Isa* incision superius apicalis, *Is* incision superius Analyse des Bewegungsausmaßes des Apex anhand prä- und posttherapeutischer Fernröntgenseitenbilder. Es wurden sagittale und vertikale Bewegungen bestimmt (mm). *ANS* „anterior nasal spine“, *PNS* „posterior nasal spine”, *Isa* „incision superius apicalis”, *Is* „incision superius”

**Fig. 4:**
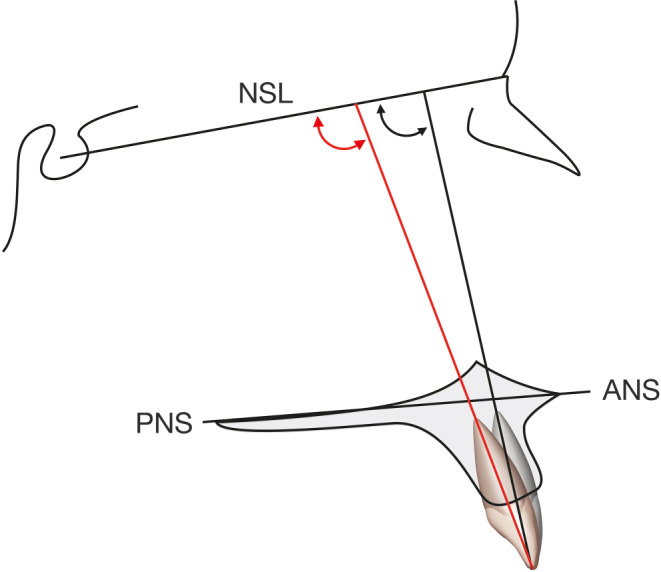
Analysis of torque change for the maxillary central incisors between pretreatment (*black line*) and posttreatment (*red line*) lateral cephalogram relative to the anterior base of the skull and the maxillary base plane Analyse der Torqueveränderung der zentralen Oberkieferinzisiven zwischen prä- (*schwarze Linie*) und posttherapeutischem (*rote Linie*) Fernröntgenseitenbild zur vorderen Schädelbasis und der Oberkiefergrundebene

The torque change resulting from treatment as well as the apical movement achieved were assessed by comparing the pre- and posttreatment lateral cephalograms. The two X‑rays were overlaid so that the anatomical structures corresponded as closely as possible. In order to assess the degree of displacement of the upper central incisors, two different measurements were acquired: (1) assessment of the sagittal and horizontal apical movement (Fig. 3) and (2) the torque change to the upper central incisors that was achieved (Fig. 4).

### Statistics

Descriptive and inferential statistics were calculated using IBM SPSS Statistics 25 (IBM Corp., Armonk, NY, USA). Cephalometric measurements and relative root-to-crown ratios were presented with mean, standard deviation (SD), range (minimum to maximum), median, and interquartile range. Since most of the measurements showed deviation from the assumption of normality and due to the sample size nonparametric inferential methods were applied. Differences between the patient groups were assessed by the Mann–Whitney U test, whereas difference between pre- and posttreatment were examined using the Wilcoxon signed-rank test. Kruskal–Wallis testing was applied to test for differences in rRCR between the different teeth in each group separately. The Pearson correlation was applied to test the relationship between root resorption and apical movements and between torque changes of the maxillary incisors and root resorption. The level of significance was set at *p* < 0.05.

## Results

**Fig. 5:**
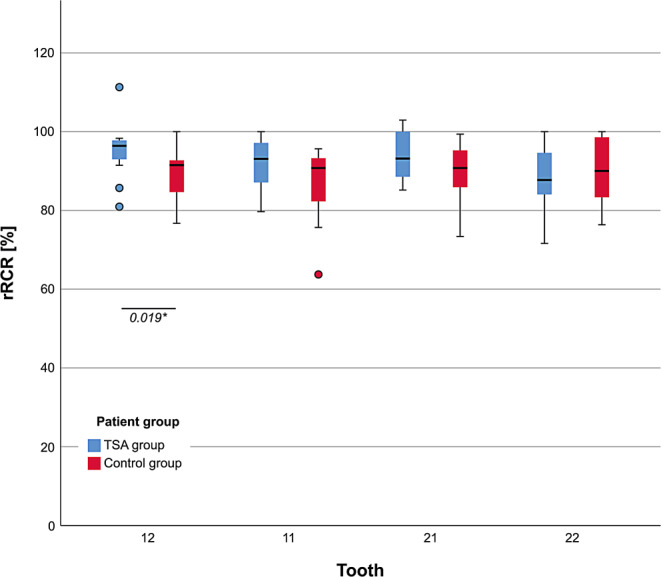
Relative change in root–crown ratio (rRCR) for tooth 11, 12, 21, 22, for torque-segmented archwire (TSA) treated and conventionally orthodontic treated patients. Pairwise comparisons were done using the Mann–Whitney U test (*asterisk* *p* < 0.05). For the comparison within each patient group the Kruskal–Wallis test was applied (*p* < 0.05) Relative Änderung des Wurzel-Kronen-Verhältnisses (rRCR) für die Zähne 11, 12, 21 und 22 bei mit TSA (Torquesegmentbogen) sowie konventionell orthodontisch behandelten Patienten. Paarweise Vergleiche wurden mit dem Mann-Whitney-U-Test durchgeführt (*Asterisk* *p* < 0,05), für den Vergleich innerhalb der Gruppen wurde der Kruskal-Wallis-Test angewendet (*p* < 0,05)

Based on a pretreatment tooth length of 100%, the mean relative root–crown ratio (rRCR) after completion of the orthodontic treatment with the TSA was 93% and in the orthodontic-treated control group 89%. The difference between the groups was significant (*U* = 3208, *p* = 0.014; Table 1). The average rRCR for the individual incisors in the TSA group ranged from 89 to 95% and in the control group from 87 to 90% (Table 1, Fig. 5). The individual teeth (tooth 12, 11, 21, and 22) did not differ significantly within both groups (Kruskal–Wallis test; TSA group: *H* = 7.512, df = 3, *p* = 0.057; control group: *H* = 1.373, df = 3, *p* = 0.712). But a significant difference was found for tooth 12 between TSA and the control group (92 vs. 96; *U* = 236.0, *p* = 0.019; Table 1, Fig. 5). The median rRCR in the control group was smaller than in the TSA group (Fig. 5).

**Table 1:** Comparison between the torque-segmented archwire (TSA) and control group concerning the amount of movement of the apices of maxillary incisors in the sagittal and vertical direction relative to the ANS-PNS plane and the relative root–crown ratio (rRCR). Number of patients (*N*), mean, standard deviation (SD), range (minimum and maximum), median and interquartile range (IQR) were reported. U statistics and significance (*p*) were given for pairwise comparisons using the Mann–Whitney U test Vergleich zwischen TSA(Torquesegmentbogen)- und Kontrollgruppe bezüglich der sagittalen und vertikalen Apexbewegung der Oberkieferfrontzähne und des relativen Wurzel-Kronen-Verhältnisses (rRCR). Anzahl der Patienten (*N*), Mittelwert, Standardabweichung (SD), Bereich (Minimum, Maximum), Median und das Interquartilsabstand (IQR) wurden dokumentiert. Für die paarweisen Vergleiche mit dem Mann-Whitney-U-Test wurden U‑Statistik und *p*-Wert gezeigt

		Patient group												Mann–Whitney U test	
		TSA group (*N* = 18)						Control group (*N* = 18)							
		*N*	Mean	SD	Range	Median	IQR	*N*	Mean	SD	Range	Median	IQR	*U*	*P*
Retrusion/protrusion (sagittal apex movement, mm)	Retrusion	14	−2.8	1.3	−5.2 to −0.9	−2.4	[−3.2; −2.0]	9	−1.7	0.7	−2.5 to −0.5	−2.0	[−2.0; −1.5]	33.0	0.062
	No movement	0	–	–	–	–	–	2	–	–	–	–	–	–	–
	Protrusion	4	1.0	0.7	0.4 to 2.0	0.7	[0.5; 1.4]	7	2.4	1.4	0.5 to 4.5	2.0	[1.5; 3.5]	6.0	0.164
	Total	18	−1.9	2.0	−5.2 to 2.0	−2.2	[−2.7; −0.9]	18	0.1	2.2	−2.5 to 4.5	−0.2	[−2.0; 1.5]	83.0	0.012*
Extrusion/intrusion (vertical apex movement, mm)	Intrusion	5	−1.1	0.7	−2.4 to −0.6	−0.9	[−0.9; −0.9]	2	−1.0	0.0	−1.0 to −1.0	−1.0	[−1.0; −1.0]	8.0	0.381
	No movement	0	–	–	–	–	–	2	–	–	–	–	–	–	–
	Extrusion	13	1.5	0.8	0.4 to 3.4	1.2	[0.9; 1.7]	14	1.8	1.0	0.5 to 4.0	1.5	[1.0; 2.3]	74.0	0.430
	Total	18	0.7	1.4	−2.4 to 3.4	1.0	[−0.6; 1.6]	18	1.3	1.3	−1.0 to 4.0	1.3	[0.5; 2.0]	134.0	0.389
rRCR, %	Tooth 11	18	92	6	80 to 100	93	[87; 97]	18	87	8	64 to 96	91	[82; 93]	218.0	0.079
	Tooth 12	18	95	6	81 to 111	96	[93; 98]	18	90	7	77 to 100	92	[85; 93]	236.0	0.019*
	Tooth 21	18	94	6	85 to 103	93	[89; 100]	18	90	7	73 to 99	91	[86; 95]	213.0	0.111
	Tooth 22	18	89	7	72 to 100	88	[84; 95]	18	90	9	76 to 100	90	[83; 99]	136.5	0.424
	Central incisors	36	93	6	80 to 103	93	[88; 97]	36	88	8	64 to 99	91	[83; 94]	441.5	0.020*
	Lateral incisors	36	92	7	72 to 111	94	[86; 97]	36	90	8	76 to 100	91	[84; 98]	573.5	0.401
	Total	72	93	6	72 to 111	93	[87; 97]	72	89	8	64 to 100	91	[84; 95]	3208.0	0.014*

Concerning the incidence of root resorption, 89% (TSA group) and 94.4% (control group) of the teeth examined showed resorptions (Table 2). In the TSA group 8/72 (11.1%) teeth were not affected with posttreatment root resorption, in the control group only 5.6%. The severity of the resorptions was predominantly in the light to moderate range (55.6% and 30.6% within the TSA group and 48.6% and 33.3% in the control group). Severe resorption was present in 2.8% of the TSA group and 12.5% of the control group.

**Table 2:** Severity of root resorption (RR). Crosstabulation of tooth and patient group with levels of severity of root resorption classified according to relative root–crown ratio (rRCR) Schweregrad der Wurzelresorption (RR). Kreuztabelle aus Zahn und Patientengruppe mit dem Niveau des Schweregrades der Wurzelresorption entsprechend des relativen Wurzel-Kronen-Verhältnisses (rRCR)

Severity of RR		12		11		21		22		Total	
		TSA group	Control group	TSA group	Control group	TSA group	Control group	TSA group	Control group	TSA group	Control group
No RR (rRCR ≥100%)	No. of teeth	1	1	1	0	5	0	1	3	8	4
	%	5.6	5.6	5.6	0.0	27.8	0.0	5.6	16.7	11.1	5.6
Slight RR (90 ≤ x < 100)	No. of teeth	15	9	10	10	8	10	7	6	40	35
	%	83.3	50.0	55.6	55.6	44.4	55.6	38.9	33.3	55.6	48.6
Moderate RR (80 ≤ x < 90)	No. of teeth	2	6	6	6	5	6	9	6	22	24
	%	11.1	33.3	33.3	33.3	27.8	33.3	50.0	33.3	30.6	33.3
Severe RR (<80)	No. of teeth	0	2	1	2	0	2	1	3	2	9
	%	0.0	11.1	5.6	11.1	0.0	11.1	5.6	16.7	2.8	12.5
Total	No. of teeth	18	18	18	18	18	18	18	18	72	72
	%	100.0	100.0	100.0	100.0	100.0	100.0	100.0	100.0	100	100

*TSA* torque-segmented archwire

Evaluation of the lateral cephalograms showed that the main directions of movement of the apices of the upper central incisors were retrusion and extrusion (Table 1). The median sagittal apex movement of the total TSA group (−2.2 mm) was statistically significant larger than that of the control group (median: −0.2 mm; *U* = 83.0, *p* = 0.012), whereas there was no statistical difference in vertical apex movement (*U* = 134.0, *p* = 0.389; Table 1).

Regarding the axial position of the front teeth, the median of the front tooth axis (1_NSL) significantly increased in the TSA group from 98.2 to 102.5° (*Z* = −2.286, *p* = 0.022; Table 3). In the control group, no significant change in the axial position (*Z* = −1.613, *p* = 0.107) was observed (Table 3).

**Table 3:** Magnitude of torque of maxillary central incisors relative to the anterior base of the skull and the ANS-PNS plane for both patient groups. Number of patients (*N*), mean, standard deviation (SD), range (minimum and maximum), median and interquartile range (IQR) were reported. *Z* statistics and significance (*p*) were given for pairwise comparisons of repeated measurements using the Wilcoxon signed-rank test Torqueausmaß zentraler Oberkieferfrontzähne in Bezug auf die vordere Schädelbasis und der ANS-PNS-Ebene für beide Patientengruppen. Anzahl der Patienten (*N*), Mittelwert, Standardabweichung (SD), Bereich (Minimum und Maximum), Median und der Interquartilsabstand (IQR) wurden dokumentiert. Für die paarweisen Vergleiche mit dem Wilcoxon-Vorzeichen-Rang-Test wurden *Z*-Statistik und *p*-Wert zusammengestellt

Patient group	Measurement	Treatment step										Wilcoxon signed-rank test	
		Pretreatment (*N* = 18)					Posttreatment (*N* = 18)						
		Mean	SD	Range	Median	IQR	Mean	SD	Range	Median	IQR	*Z*	*p*
TSA group	1_NSL [°]	96.5	7.7	80.6 to 109.4	98.2	[90.0; 102.4]	101.3	6.1	85.3 to 109.8	102.5	[98.2; 104.2]	−2.286	0.022*
	1_NL [°]	103.1	7.7	87.3 to 113.6	105.3	[95.4; 108.8]	109.4	6.8	92.7 to 124.5	110.1	[106.8; 111.9]	−3.114	0.002*
Control group	1_NSL [°]	102.8	8.2	80.0 to 115.0	104.0	[98.0; 109.0]	106.8	6.7	88.5 to 120.5	107.5	[105.5; 111.0]	−1.613	0.107
	1_NL [°]	110.1	6.9	95.0 to 121.0	110.5	[107.0; 114.0]	114.3	5.3	100.0 to 123.0	115.0	[112.0; 117.5]	−1.918	0.055

*TSA* torque-segmented archwire

**Fig. 6:**
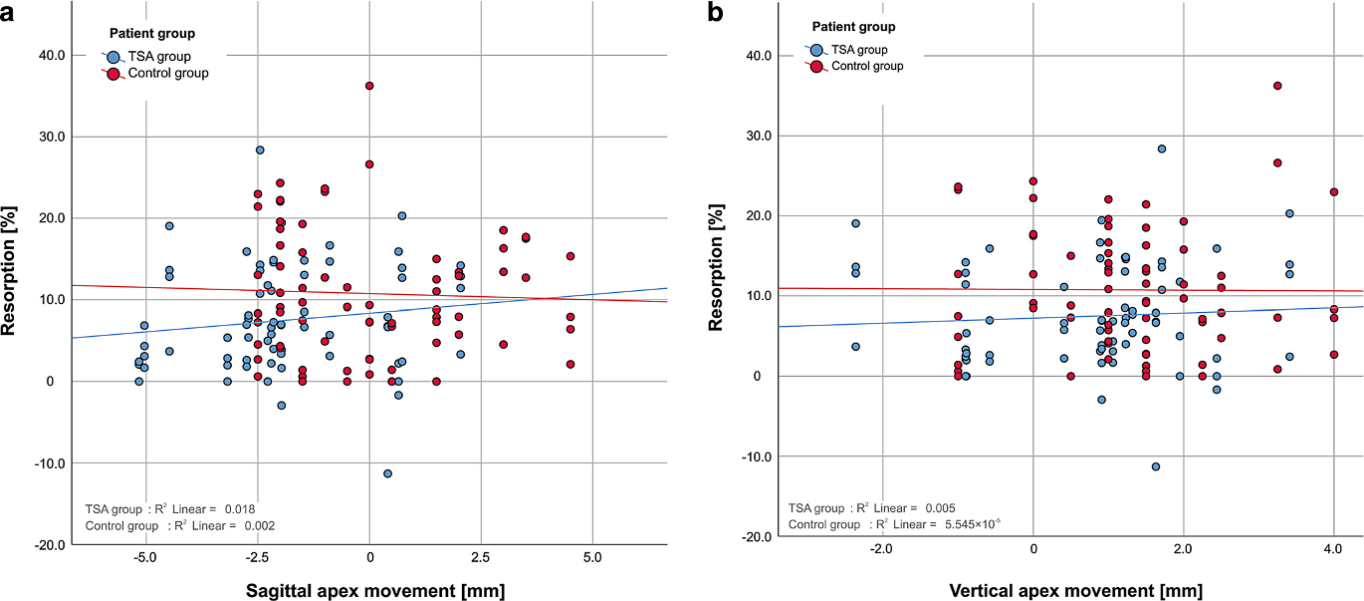
**a** Relationship between resorption of the central and lateral incisors and sagittal apex movement for both patient groups. No significant correlation between sagittal apex movement and resorption was found in either group. **b** Relationship between resorption of the central and lateral incisors and vertical apex movement for both patient groups. No significant correlation between vertical apex movement and resorption was found in either group. *TSA* torque-segmented archwire **a** Beziehung zwischen Wurzelresorption der zentralen und lateralen Schneidezähne und der sagittalen Apexbewegung in beiden Patientengruppen. Eine signifikante Korrelation zwischen sagittaler Apexbewegung und Resorption ließ sich in beiden Gruppen nicht feststellen. **b** Beziehung zwischen Wurzelresorption der zentralen und lateralen Schneidezähne und der vertikalen Apexbewegung in beiden Gruppen. Eine signifikante Korrelation zwischen vertikaler Apexbewegung und Resorption konnte in beiden Gruppen nicht festgestellt werden

**Fig. 7:**
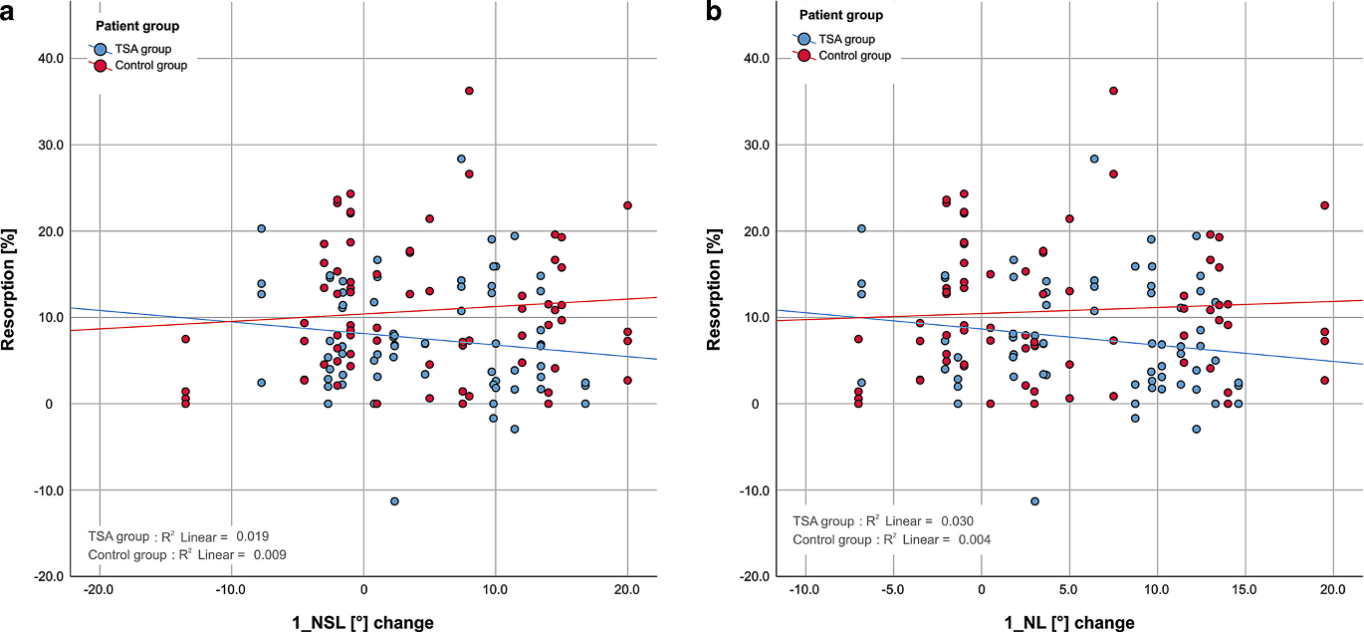
**a** Relationship between resorption of the central and lateral incisors and 1_NSL change for both patient groups. No significant correlation between 1_NSL change and resorption was found in either group. **b** Relationship between resorption of the central and lateral incisors and 1_NL change for both patient groups. No significant correlation between change of 1_NL and resorption was found in either group. *TSA* torque-segmented archwire **a** Beziehung zwischen Wurzelresorption der zentralen und lateralen Schneidezähne und 1_NSL-Änderung in beiden Patientengruppen. Eine signifikante Korrelation zwischen 1_NSL-Änderung und Resorption konnte in beiden Gruppen nicht festgestellt werden. **b** Beziehung zwischen Wurzelresorption der zentralen und lateralen Schneidezähne und 1_NL-Änderung in beiden Gruppen. Eine signifikante Korrelation zwischen Änderung des 1_NL und Resorption ließ sich in beiden Gruppen nicht feststellen

Pearson’s correlation coefficients for resorptions at maxillary incisors and horizontal direction of apical movement were *r* = 0.136 (*p* = 0.255) for the TSA group and *r* = 0.042 (*p* = 0.727) for the control group and were not statistically significant (Fig. 6a). For resorptions at maxillary incisors and vertical direction of apical movements the Pearson’s correlation coefficients were *r* = 0.067 (*p* = 0.575) for the TSA group and *r* = −0.007 (*p* = 0.950) for the control group and were not statistically significant (Fig. 6b). Both correlation coefficients show that there is no linear correlation between horizontal or vertical direction of movement of the apex and the amount of resorption of upper incisors. A correlation between resorption at maxillary incisors and change in both toot axis 1_NSL (Fig. 7a) and 1_NL (Fig. 7b) was not evident in the TSA group (1_NSL: *r* = 0.139, *p* = 0.243; 1_NL: *r* = 0.172, *p* = 0.148) nor in the control group (1_NSL: *r* = 0.094, *p* = 0.430; 1_NL: *r* = 0.066, *p* = 0.584). The correlation coefficients show that there is no linear correlation between change in tooth axis 1_NSL or 1_NL and the amount of resorption of upper incisors. The *p*-values calculated were not statistically significant, given a level of significance of *p* < 0.05.

The effect of treatment duration on the amount of resorption of upper incisors was not significant in either patient group. Pearson’s correlation coefficient was *r* = 0.195 (*p* = 0.101) for the TSA group and *r* = 0.068 (*p* = 0.571) for the control group.

## Discussion

The accurate and controlled biomechanics of the TSA [47, 55] makes it possible to exert defined moments on the teeth and thereby achieve clinically very efficient results [3, 47]. The results also show that within a 1_NSL range of 85.3–109.8° torque adjustment with defined moments even with preactivated torque wires remains difficult. In addition, biomechanical and biological factors can play a role. Torque can be applied with stainless steel arch wires as well as the TSA. The inconsistency of data between the two methods shows that clinically the adjustment of the M/F ratio depends on other factors. The application of the TSA simplifies clinical application of torque. However prospectively, for better M/F ratio control different moments should be used. For bodily retraction, moments in the range of 15–20 Nmm are reported [5, 9, 14], whereas for controlled tipping lower moments can be applied. Further clinical studies must show whether this will lead to a better adjustment of the M/F ratio.

In most of the studies on continuous forces and moments, there was nevertheless a loss of force or moment during the progress of the treatment [1, 30, 36–38], although the study design in these studies is generally a matter of discussion. The torque we used on the four maxillary incisors with an overall magnitude of 15–20 Nmm [55] is within the physiological range of the ideal level of torque [5, 9, 14]. This torque range is discussed in order to minimize resorptions at the apical root tip [10, 12, 13, 18, 19, 21, 24, 42, 44, 48].

The methodology to assess the resorption rate during orthodontic treatment applied in our study is known from the literature and well established [15, 16, 26]. It allows use of X‑rays, which are recorded in the diagnostic routine of an orthodontic treatment. Therefore, it avoids additional radiation exposure of the patient. As panoramic radiographs are accompanied by perspective distortions, the use of the relative root-to-crown ratio (rRCR) allows one to calculate a decresase in root length [15]. With the assumption of a constant crown length, these distortions are compensated. A much more detailed analysis of root resorptions is only possible using three-dimensional radiographic techniques like cone-beam computed tomography [4, 43]. Unfortunately, these X‑ray techniques are accompanied by high additional radiation exposure and have a strong indication.

The average loss of apical root length (rRCR) of 7% in the TSA group and 11% in the control group recorded in the present study can be regarded as not clinically relevant. The median rRCR of the maxillary incisors was significant larger in the TSA group as compared to the control group. Therefore, root resorption was less expressed in the TSA group although the difference was only 2%. In our study, no significant differences were found between the maxillary incisors within one group and between the groups except tooth 12. Our results for the maxillary central incisor coincide with the amount of resorption recorded in most other studies that analyzed the extent of root resorption found on upper central incisors after orthodontic treatment with fixed appliances [7, 12, 15, 22, 29, 32, 40, 43, 50]. In the present study, however, no statistically significant correlation was noted for resorptions and treatment-related apical movement in the horizontal or vertical direction. On a cautionary note, however, it should be mentioned that the apical movement distances were calculated on the lateral cephalograms, for which the interval between pre- and posttreatment X‑rays does not always coincide precisely with the period when the TSA is in place. Hence, a treatment-related unwanted jiggling effect in other treatment phases cannot be ruled out as an important contributory factor to root resorption. The major advantage of utilizing the superelasticity of these materials for the practitioner is that, even if the wire is excessively activated, the torque acting on the tooth remains within physiological limits. Hence, rectangular wires can be used even at an early stage; these allow better control of tooth movement, especially during torque application [52]. An evaluative comparison of the different types of moment application is only possible if a controlled comparative test is done in the same individual, e.g., by applying a split-mouth design, but this is clinically not realistic.

No correlation between root resorption and incisor axis change was found in our study. A review of the literature shows that there are contradictory findings concerning this correlation [12, 32, 40, 41]. Multifactorial influences on the amount of root resorption are discussed. Due to the variety of study designs, no consistent statement is found in the literature. Most of the authors have not found any correlation between the difference of incisor inclination and root resorption [12, 32, 40, 41].

Although we found no significant correlation (*p* ≥ 0.101) between duration of torque application and root resorption, it seems reasonable to assume that the amount and the duration of the torque application have an influence on the grade of root resorption [10, 45]. A longer treatment duration is associated with significantly higher root resorption rates for maxillary central incisors [20, 25, 49]. Only a few studies deal with the time dependence of treatment-related root resorption when continuous forces are applied by means of NiTi components [10, 35]. Although the correlation was not significant in our study, there was a trend towards an increased risk of root resorption with a longer treatment duration.

The results of this study show no correlation between vertical and sagittal apex movement and root resorption. This correlation is controversially discussed in the literature [7, 12, 15, 22, 29, 32, 43, 50]. Factors like study design, patient number and methodology should be discussed. In 4 patients from the TSA group and 7 patients from the control group, a sagittal apex movement of 1 or 2 mm was observed, respectively. This indicates an uncontrolled tipping of the incisors. This effect arises due to differences in root geometry and nonideal force–moment application.

The results of this retrospective study should be validated with further prospective studies with a larger patient group and measurements of the actual torque moment acting on the teeth, to gain more knowledge about the ideal level of moment for continuous torque application, and to obtain more clear-cut results.

## Conclusion

- Use of the torque-segmented archwire (TSA) for continuous torque application on the maxillary anterior teeth leads to slight, clinically nonrelevant apical root resorption.
- The incisors axis in the TSA group was on average significantly improved. Independently, though defined moments were applied, the adjustment of the M/F ratio was still problematic. In order to achieve a better adjustment of the M/F ratio, the application of different moments is advised.
- No differences were found between the amount of apical root resorption of maxillary lateral and central incisors in the TSA and control groups.
- Between the TSA and control group significant differences in the relative root–crown ratio (rRCR) were found for tooth 12, the central incisors, and for all maxillary incisors. Although root resorption in the TSA group was less pronounced, the difference between the groups was only 2%.
- There is no correlation between root resorption and treatment duration, the distance of vertical and sagittal movement of the apex and the change in inclination of the incisal axis relative to the anterior base of the skull.
